# Noradrenergic, Serotonergic and Dopaminergic Modulation in AD‐Brexpiprazole

**DOI:** 10.1002/alz70857_098884

**Published:** 2025-12-24

**Authors:** Elizabeth A. Crocco

**Affiliations:** ^1^ University of Miami Miller School of Medicine, Miami, FL, USA

## Abstract

**Background:**

Neuropsychiatric symptoms and agitation in AD are common. Evidence suggests that non‐cognitive symptoms can lead to a more rapid progression of the illness. Additionally, they are closely aligned with neurotransmitter dysregulation that originates in the neuromodulory subcortical nuclei (NSS), an area well characterized as dysfunctional in early AD.

**Method:**

Data suggests that the pathophysiology of agitation in AD constitutes an imbalance within the prefrontal cortex and subcortical brain regions resulting from noradrenergic (NA) hyperactivity, serotonin (5‐HT) system deficits and increases in striatal dopamine (DA).

**Result:**

Brexpiprazole, a novel atypical antipsychotic is the first FDA approved drug for Agitation in AD and is classified as a serotonin‐dopamine activity modulator (SDAM). Additionally, it is a potent antagonist of noradrenaline alpha 1B/2C receptors. It has demonstrated a reduction in agitation in AD subjects.

**Conclusion:**

This talk will discuss brexpiprazole as well as comparisons to similar treatments that act as modulators to the NSS system. It will discuss the advantages, side effects, potential risks and implications for further research to reduce symptom burden and potentiate disease modification.

